# Polymer-metal hybrid transparent electrodes for flexible electronics

**DOI:** 10.1038/ncomms7503

**Published:** 2015-03-19

**Authors:** Hongkyu Kang, Suhyun Jung, Soyeong Jeong, Geunjin Kim, Kwanghee Lee

**Affiliations:** 1 School of Materials Science and Engineering, Heeger Center for Advanced Materials and Research Institute for Solar and Sustainable Energies, Gwangju Institute of Science and Technology, Gwangju 500-712, Republic of Korea

## Abstract

Despite nearly two decades of research, the absence of ideal flexible and transparent electrodes has been the largest obstacle in realizing flexible and printable electronics for future technologies. Here we report the fabrication of ‘polymer-metal hybrid electrodes’ with high-performance properties, including a bending radius <1 mm, a visible-range transmittance>95% and a sheet resistance <10 Ω sq^−1^. These features arise from a surface modification of the plastic substrates using an amine-containing nonconjugated polyelectrolyte, which provides ideal metal-nucleation sites with a surface-density on the atomic scale, in combination with the successive deposition of a facile anti-reflective coating using a conducting polymer. The hybrid electrodes are fully functional as universal electrodes for high-end flexible electronic applications, such as polymer solar cells that exhibit a high power conversion efficiency of 10% and polymer light-emitting diodes that can outperform those based on transparent conducting oxides.

## Abstract (web summary)

Building printable electronics demands flexible and transparent electrodes that can be fabricated on plastic substrates. Here, Kang *et al.* report a polymer-metal hybrid electrode that has a small bending radius of <1 mm, a sheet resistance of 10 Ω sq^−1^ and a transparency in the visible range of >95%.

## Graphical abstract

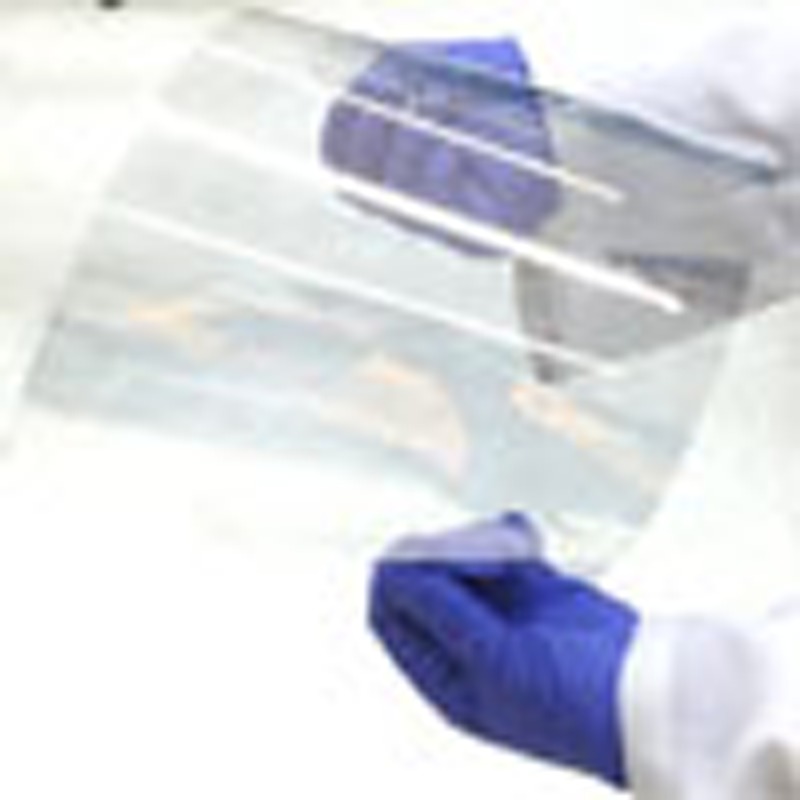

Future ubiquitous electronic devices require versatile features, such as being lightweight and exhibiting portability, wearability and flexibility with a superior mechanical stability^1^,^2^. In addition, the fabrication of such devices is based on solution processing methods of organic materials, known as ‘printable electronics’, which ultimately integrate high-throughput, roll-to-roll processing^3^,^4^. This new emerging technology inevitably demands high-standard flexible and transparent electrodes (FTEs) fabricated on plastic substrates. Recently, there have been intense research efforts to develop new FTEs, such as conducting metal oxides^5^,^6^, conducting polymers (CPs)^7^,^8^, carbon nanotubes^9^,^10^, graphene^11^,^12^, metal nanowires^13^,^14^ and hybrid electrodes^15^,^16^. Despite the impressive progress made in the past decades, however, further enhancement in the performance of the FTEs fabricated on ‘flexible plastic substrates’ is still required to meet the standard specifications, namely a transmittance (*T*) ≥90% in the visible range and a sheet resistance (*R*_sh_) ≤10 Ω sq^−1^.

The fabrication of ultrathin metals with a thickness (*δ*≤10 nm) smaller than the penetration depth (*δ*_p_)—which allow for visible light transmission while still being capable of metallic transport along the lateral direction—has been considered an emerging technology for realizing ideal FTEs^17^,^18^,^19^,^20^. In general, the vacuum deposition of conventional metals on transparent substrates (glass or plastics) is governed by a ‘random-nucleation’ process^17^,^18^,^19^,^20^,^21^. Thermally evaporated (or sputtered) metal droplets migrate easily and aggregate randomly on substrate surfaces because of the surface energy mismatch between the two materials. This nucleation process causes three-dimensional metal-island growth on the substrates and results in a discrete granular morphology in the thin-film regime (*δ*≤10 nm). Therefore, the percolation threshold (*τ*_p_) of metal films, in which the metal islands are completely interconnected throughout the deposited films, will occur in a thicker layer than the *δ*_p_ of typical metals (for example, *δ*_p_≈15 nm at 550 nm for Ag) and does not allow for visible light transmission through the metal films (that is, *τ*_p_≥*δ*_p_).

One method to overcome this problem is to introduce a ‘nucleation-inducing seed layer’ (abbreviated herein as ‘nucleation inducer’) on top of the substrates, such as metal seeds^17^, metal oxides^18^, organic small molecules^19^ or a combination thereof^20^. These nucleation inducers are expected to provide dense nucleation centres and suppress the growth of large metal islands during film deposition, which would thereby reduce *τ*_p_ to below *δ*_p_. Although attempts have led to considerable improvement in the quality of ultra-thin, metal-based FTEs, there is still almost no ideal nucleation inducer that enables ultrathin metals to be smoothly formed on flexible plastic substrates and to be fully functional for high-end flexible electronic applications, which require the high-standard FTE specifications together with the smooth surface morphology. Moreover, conventional nucleation inducers that require tedious vapour deposition^19^ and/or high-temperature annealing processes^20^ are not compatible with high-speed, roll-to-roll processing on large-area plastic substrates. Thus, developing a new method of inducing well-ordered metal nuclei on plastic substrates has been a significant challenge.

Here, we present an innovative method to create uniform, strong metallization nuclei on plastic substrates in which the nucleation centres are densely distributed over atomic-scale distances. We succeed in fixing metal droplets on functional amine groups via coordination-bond interactions by introducing a solution-processed, nonconjugated polyelectrolyte equipped with functional amine groups on top of the plastic substrates. With the successive deposition of a facile anti-reflective coating using a CP on top of ultra-thin metals, we obtain high-standard FTEs that exhibit outstanding performances, including a bending radius <1 mm, a visible-range *T*>95% and a *R*_sh_<10 Ω sq^−1^. Thus, polymer devices using our FTEs exhibit excellent device performance. Polymer solar cells (PSCs) that incorporate our FTEs exhibit a high power conversion efficiency (*η*) of 10% and the polymer light-emitting diodes (PLEDs) outperform those based on any existing FTEs, including transparent conducting oxides.

## Results

### FTE structure and fabrication

**Figure 1:**
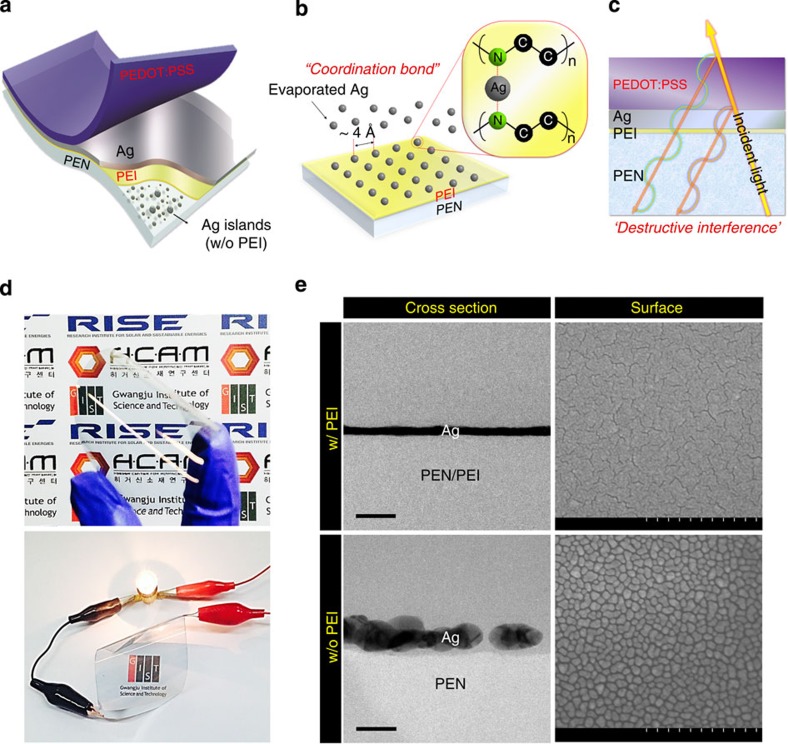
Schematic illustration of the PAP electrode. (**a**) Flexible PAP electrode consisting of the ultra-thin Ag film between the PEI and PEDOT:PSS supporting layers. (**b**) Conceptual diagram for the growth mechanism of the Ag film with the PEI nucleation inducer. (**c**) Conceptual diagram for the destructive interference in the Ag film with the PEDOT:PSS anti-reflective layer. (**d**) Images of the large-area, flexible PAP. (**e**) Cross-sectional and surface morphology images of the PEI-Ag and bare-Ag electrodes taken using transmission electron microscopy (TEM) and scanning electron microscopy (SEM), respectively. Scale bars, left 50 nm, right 500 nm.

A schematic diagram of our FTE structure is shown in Fig. 1a. The electrode consists of a Ag layer sandwiched between two different polymers, polyethyleneimine (PEI) and poly(3,4-ethylenedioxythiophene):poly(styrenesulfonate) (PEDOT:PSS), denoted as ‘PEI/Ag/PEDOT:PSS (PAP)’. We specially introduced the two polymer systems, PEI as a nucleation inducer and PEDOT:PSS as an anti-reflection layer, to synergistically enhance the optoelectronic performance of the PAP electrode, as discussed in more detail below (Fig. 1b,c). Therefore, our proposed PAP electrode is defined as a ‘polymer-metal hybrid electrode’, which is compatible with continuous roll-to-roll processing on plastic substrates and also is applicable for foldable electronic systems because of the low-temperature solution processability and flexibility of the two polymers. In addition, we confirmed that our PAP electrode can be widely expandable to a flexible PAP sheet with a large area that has comparable performance to the small PAP electrode, which demonstrates that the PAP electrode has a scalable electrode architecture to realize large-area electronic systems (Fig. 1d and Supplementary Fig. 1).

The key component material of this work is the PEI nucleation inducer shown in Fig. 1b. In our previous works^22^,^23^,^24^, this material, in which functional amines develop molecular dipoles and shift the work function of the metal electrodes, was successfully introduced as an efficient cathode interfacial layer for inverted PSCs. When we coated plastic substrates with approximately 5 nm of PEI from solution, the Ag deposition was observed to be strongly influenced by this PEI layer. The functional amine groups of PEI act as a ligand and donate an unshared electron pair to the Ag atoms to create a coordinate covalent bond (Fig. 1b and Supplementary Fig. 2)^25^,^26^. Therefore, the initially deposited Ag atoms are immobilized by the formation of a coordination complex with the amine groups of the PEI-coated substrate, as schematically described in Fig. 1b. This coordination reaction results in uniformly, densely distributed Ag nuclei over the entire surface of the substrate without suffering any random Ag migration and aggregation that is responsible for granular metal-island growth.

In particular, the essence of this new architecture arises from the density of the Ag nuclei formed by these coordination bonds. Given that the atomic spacing between the amine groups in the PEI repeating unit is approximately 4 Å, the resulting Ag nuclei may be extremely dense at these angstrom-scale intervals. Moreover, because the nature of the coordination bonds assures a strong bond between the initial Ag atoms and the amines, the following Ag deposition is centred around the densely, uniformly distributed Ag nuclei. Then, the film deposition continues with two subsequent film growth steps (that is, coalescence and thickness growth), which results in smooth Ag films with an ultra-thin thickness less than *δ*_p_ (approximately 15 nm in the visible range).

### FTE performance

**Figure 2:**
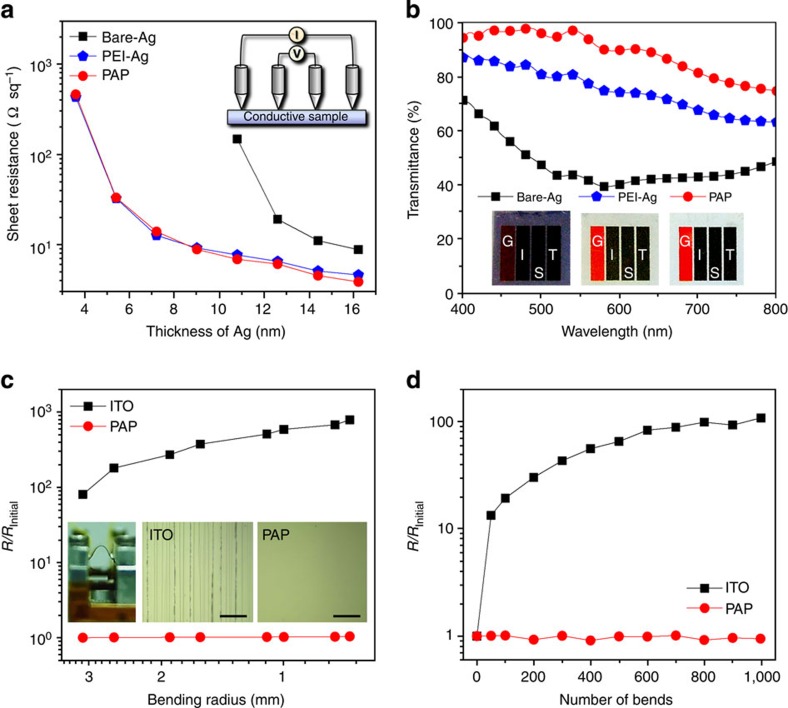
Performance of the PAP electrode. (**a**) Variations in *R*_sh_ of the bare-Ag (black square), PEI-Ag (blue pentagon) and PAP (red circle) electrodes as a function of Ag thickness. The PEI layer efficiently reduces the *τ*_p_ of the Ag films to less than 10 nm via a coordination bond-induced nucleation process. The inset shows the four-point probe system used to measure *R*_sh_. (**b**) Optical transmittance of the bare-Ag (black square), PEI-Ag (blue pentagon) and PAP (red circle) electrodes. Through destructive interference, the PEDOT:PSS layer leads to a 20% greater transmittance of the PAP electrode than that of the PEI-Ag electrode. The inset shows images of the electrodes located on top of our school logos. (**c**,**d**) Increase in resistance versus the bending radius (**c**) or number of bends (**d**) for the flexible PAP (red circle) and ITO (black square) electrodes on the PEN substrates. The insets show images of the bending test machine used (left) and the optical microscope images of the ITO and PAP electrodes after bending (right). Scale bar, 100 μm.

Direct evidence of the PEI effect can be obtained by comparing the cross-sectional transmission electron microscopy (TEM) and surface scanning electron microscopy (SEM) images for the substrate/PEI/Ag electrodes (PEI-Ag) and substrate/Ag electrodes (bare-Ag) shown in Fig. 1e. The PEI-Ag electrode exhibits a smooth and continuous morphology, as expected, whereas the bare-Ag electrode exhibits a discrete granular morphology with a thicker but disconnected Ag film cross-section. We confirmed that the root-mean-square surface roughness value (0.23 nm) of the PEI-Ag electrode is much smaller than that (2.61 nm) of the bare-Ag electrode because the PEI layer prevents the random migration and aggregation of the deposited Ag atoms (Supplementary Fig. 3). Thus, the PEI-Ag electrodes are highly conductive with an extremely low *R*_sh_≈9 Ω sq^−1^ at *δ*≈9 nm, whereas the bare-Ag electrodes are not electrically connected (*R*_sh_≈∞) below *δ*≤10 nm (Fig. 2a). Furthermore, the transmittance of the PEI-Ag electrode is increased considerably over the entire visible range (*T*≈80% at 550 nm) compared with that of the bare-Ag electrode, which is opaque due to the irregular thickness of the Ag islands (*T*≈40% at 550 nm; Fig. 2b)^19^,^20^,^27^.

However, despite the considerable enhancement in the *T*-spectrum, the PEI-Ag electrodes still do not meet the demanding requirement (*T*≥90%) for FTEs of future optoelectronic devices because substantial portions of the incident light are still reflected by the Ag film. To solve this problem, we used the PEDOT:PSS layer as an anti-reflection layer to minimize the light reflection of the Ag film (Fig. 1c). The PEDOT:PSS layer was spin-coated from solution with a thickness of approximately 50 nm on top of the PEI-Ag electrode, thereby completing our novel ‘polymer-metal hybrid electrode’ with a structure of PEI/Ag/PEDOT:PSS that has a smooth surface morphology (root-mean-square≈0.46 nm) compatible with high-end thin-film device applications (Supplementary Fig. 3). The alternating layers of the Ag film with a low refractive index (*n*_Ag_≈0.12 at 550 nm) and the PEDOT:PSS layer with a high refractive index (*n*_PEDOT:PSS_≈1.44 at 550 nm) shift the phase of the incident light at their interfaces and then induce destructive interference that reduces the reflection of the Ag film (Fig. 1c and Supplementary Fig. 4).

According to the theoretical models for anti-reflection coatings^28^, the thickness of the PEDOT:PSS layer is expected to significantly impact the magnitude of the destructive interference. Therefore, through optical simulations based on a transfer matrix method^29^,^30^, we calculated the optimum thickness of the PEDOT:PSS to be approximately 50 nm (Supplementary Fig. 5), which is consistent with the experimental results. Because PEDOT:PSS is a well-known CP^9^,^10^ that is highly conductive and transparent, in contrast to insulating or semiconducting polymers, the optimum thickness (~50 nm) of the PEDOT:PSS layer is acceptable for our new FTE architecture because it does not incur any optoelectronic losses. After coating PEDOT:PSS on the PEI-Ag electrode, the PAP electrode becomes more transparent than the PEI-Ag electrode (Fig. 2b). Over the visible range (that is, between 400 and 600 nm), the *T*-value of the PAP electrode increases by nearly 20% compared with that of the PEI-Ag electrode and leads to an impressive *T* of ~96% at 550 nm and a maximum *T* of ~98% at 485 nm.

In addition to the outstanding optoelectronic performances of *R*_sh_≈9 Ω sq^−1^ with *T*≥90% in the visible range, our PAP electrodes possess another important advantage, namely, mechanical flexibility and durability under bending stress. The resistance changes of the electrodes on the flexible poly(ethylene naphthalate) (PEN) substrates were measured as a function of the bending radius, as shown in Fig. 2c. The PAP electrode was bent to a radius of approximately 0.7 mm with no corresponding increase in resistance, whereas the resistance of the indium tin oxide (ITO) electrode increased by three orders of magnitude under the same bending radius. Moreover, we confirmed that the PAP electrode exhibited an excellent mechanical stability with a nearly constant resistance after continuous bending cycles, whereas the resistance of the ITO electrode drastically increased (Fig. 2d). These results indicate that the PAP electrode is extremely flexible but does not create microscopic cracks along the direction perpendicular to that of the bending stress due to the use of flexible supporting polymers, PEI and PEDOT:PSS.

### PSC performance

**Figure 3:**
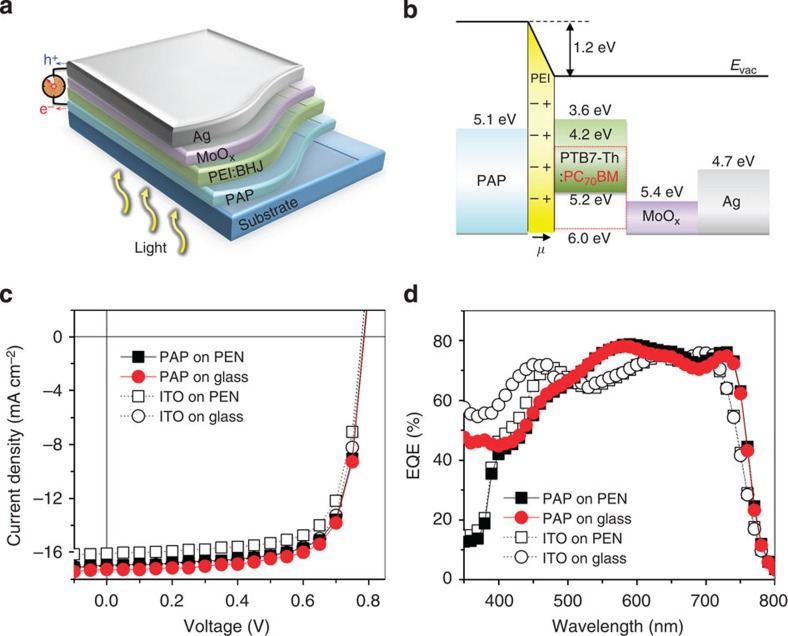
PSC applications using the PAP electrodes. (**a**) Device architecture of the PSCs, which is comprised of the PEI:PTB7-Th:PC_70_BM photoactive layer between the bottom PAP cathode and top Ag anode. (**b**) Energy level diagram of the PSCs. The self-assembled PEI layer induces a work function reduction in the PAP cathode by forming interfacial dipoles, which leads to favourable ohmic contact between the PAP and PC_70_BM. (**c**,**d**) *J-V* characteristics (**c**) and EQE spectra (**d**) of the PSCs based on different electrodes and substrates. The devices using the PAP electrodes outperform the devices using conventional ITO electrodes. Black solid square, red solid circle, black open square and black open circle represent PAP on PEN, PAP on Glass, ITO on PEN and ITO on Glass, respectively.

To evaluate the charge collection capability of the PAP electrodes, we fabricated four types of bulk heterojunction (BHJ) PSCs with an inverted structure consisting of a substrate (glass or PEN)/cathode (PAP or ITO)/photoactive layer/molybdenum sub-oxide (MoO_x_)/Ag anode. The device architecture and corresponding energy level diagram are presented in Fig. 3a,b, respectively. For the photoactive layer, we used a versatile ternary blend that was prepared by adding a small amount of the PEI solution to the BHJ solution containing the poly(thieno[3,4-b]thiophene-alt-benzodithiophene) derivative (PTB7-Th) as an electron donor^31^ and a fullerene derivative [6,6]-phenyl-C_71_-butyric acid methyl ester (PC_70_BM) as an electron acceptor. Based on our previous work^22^, the one-step solution processing of the ternary PEI:BHJ composite on the PAP electrode can form a graded bilayer with bottom-enriched PEI and top-enriched BHJ via the vertical phase separation that is induced by a surface energy difference between the hydrophilic PEI and hydrophobic BHJ materials (Supplementary Fig. 6). The self-organized PEI bottom layer can significantly reduce the work function of the PAP electrode from 5.1 to 3.9 eV by forming interfacial dipoles between the positively charged amines of the PEI and the negatively charged sulfonate anions of the PEDOT:PSS, which thereby creates favourable ohmic contacts with the lowest unoccupied molecular orbital level of the PC_70_BM for efficient inverted PSC operation (Fig. 3b)^23^,^24^.

The current density–voltage (*J-V*) characteristics of the PSCs were measured under standard 1 sun simulated solar illumination with an Air Mass 1.5 global (AM 1.5G) and an irradiation intensity of 100 mW cm^−2^. The photovoltaic parameters are summarized in Table 1. As shown in Fig. 3c, the PAP-PSCs yield high *η* values of 9.9% on PEN and 10.1% on glass. Surprisingly, those performances are even superior to those of conventional ITO-based PSCs, for which *η*≈9.5% on PEN and *η*≈9.7% on glass. Considering the nearly identical open circuit voltages (*V*_oc_) and fill factors of all of the devices, the higher *η* values of the PAP-PSCs are primarily attributed to the increased short-circuit current density (*J*_sc_; Fig. 3d). We attribute the improved photocurrent responses to a microcavity-induced light trapping effect originating from the internal reflection between the bottom PAP cathode and the top Ag anode^20^,^32^. Indeed, the absorption spectra measured under reflection mode demonstrate a greater increase in the light absorption intensity in the PAP-PSCs than in the ITO-PSCs (Supplementary Fig. 7).

### PLED performance

**Figure 4:**
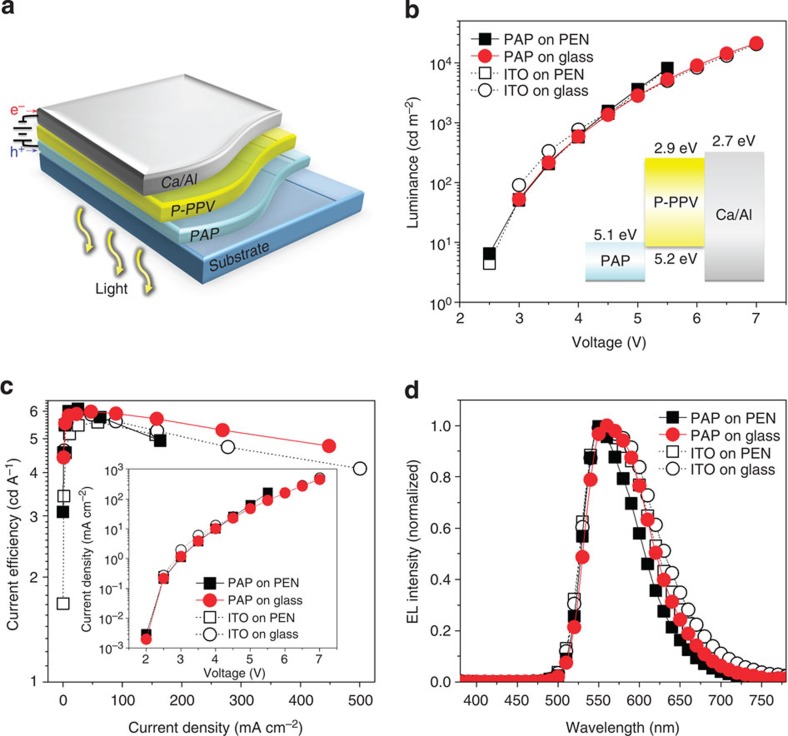
PLED applications using the PAP electrodes. (**a**) Device architecture of the PLEDs using the P-PPV emissive layer between the PAP anode and Ca/Al cathode. (**b**,**c**) *L-V* characteristics (**b**) and *C-V* characteristics (**c**) of the PLEDs. The performances of the PAP-based PLEDs are slightly greater than or comparable with those of the ITO-based PLEDs. The insets show the energy level diagram and *J-V* characteristics of the PLEDs. (**d**) Electroluminescent (EL) spectra of the PLEDs. The EL spectrum of the PAP-based device is nearly identical to that of the ITO-based device. The inset shows an image of the flexible PLEDs with the PAP electrode. Black solid square, red solid circle, black open square and black open circle represent PAP on PEN, PAP on Glass, ITO on PEN and ITO on Glass, respectively.

To assess the charge injection characteristics of the PAP electrodes, we also fabricated four PLEDs with a normal device structure of substrate (glass or PEN)/anode (PAP or ITO/PEDOT:PSS)/aryl-substituted poly(*para*-phenylene vinylene) derivative (P-PPV)/Ca/Al cathode (Fig. 4a). The luminance (*L*)-*V* characteristics, including the energy level diagram of the device and current efficiency (*C*)-*J* characteristics, which contain the *J-V* curve, are shown in Fig. 4b,c, respectively. The PAP-PLEDs also exhibit comparable or slightly superior LED characteristics compared with the ITO-based PLEDs (Table 1). The similarity in the LED parameters originates from the nearly identical *R*_sh_ values (approximately 10 Ω sq^−1^) of the PAP and ITO electrodes and from the same injection contacts (PEDOT:PSS and Ca) at their interfaces between the P-PPV and electrodes. The electroluminescent spectra also indicate that the light emission of the PAP-PLEDs is almost identical to that of the ITO-based PLEDs without any significant spectrum shifts due to the similarity in the high optical transmittances of the PAP electrodes and ITO electrodes in the visible region (Fig. 4d and Supplementary Fig. 8).

## Discussion

Our work represents a new scientific insight into the formation of well-ordered metal nuclei via a coordination reaction and a remarkable advance towards developing efficient FTEs for next-generation printable electronic systems. The synergetic combination of the nonconjugated polyelectrolyte as a nucleation inducer and the CP as an anti-reflective layer has led to a new class of polymer-metal hybrid FTEs that possess low *R*_sh_ (<10 Ω sq^−1^), high *T* (>95% at 550 nm) and extreme flexibility (bending radius<1 mm) and allows us to fabricate efficient, flexible and printable ITO-free PSCs and PLEDs that outperform ITO-based devices. Our new approach is expected to help realize printable ubiquitous optoelectronic applications and provide scientific inspiration to related research fields.

## Methods

### Material preparation

PEI (Sigma-Aldrich, 50 wt% in H_2_O) was diluted in deionized water to create a 0.3 wt% aqueous solution. The PEDOT:PSS (VPAI 4083, H.C. Starck) solution was diluted in 2-methoxyethanol at a 1:9 volume ratio to optimize the thickness of the PEDOT:PSS layer. The PTB7-Th:PC_70_BM solution was prepared by blending PTB7-Th and PC_70_BM (1:1.5 by weight) in chlorobenzene solvent with 1,8-diiodooctane additive (3% by volume) for a total concentration of 25 mg ml^−1^. The PEI:BHJ solution was obtained by adding the dilute PEI solution (0.1 wt% in 2-methoxyethanol) to the PTB7-Th:BHJ solution at a 1:9 volume ratio. The P-PPV polymer was dissolved in toluene to obtain a 0.6 wt% solution.

### Electrode fabrication and characterization

The structure of the fabricated PAP electrode was substrate/PEI/Ag/PEDOT:PSS. The pre-cleaned substrates were treated with ultraviolet–ozone. The PEI solution was spun-cast onto the substrates at 5,000 r.p.m. and air dried at 100 °C for 20 min. Ag (9 nm) was then deposited by thermal evaporation under a high vacuum (<10^−7^ Torr). Finally, the PEDOT:PSS solution was spun-cast onto the Ag film at 3,000 r.p.m. For the TEM measurement, thin specimens of the PEI-Ag and bare-Ag electrodes were obtained using a focused ion beam technique. Cross-sectional and surface images of the samples were characterized by TEM and SEM, respectively. The TEM images were obtained with a Tecnai G2 F30 S-Twin microscope operated at 300 kV, and the SEM images were acquired with a Quanta 200 FEG microscope operated at 10 kV. The *R*_sh_ values were measured using a custom-built four-point probe system with a Keithley 2,400 Source Measure Unit. Transmittance in the spectral range of 400–800 nm was measured using a Perkin-Elmer Lambda 750 UV/Vis/NIR spectrophotometer. The electrode transmittance was measured by excluding those of the PEN substrates. The optical images were obtained using an optical microscope (Axio Scope.A1, Carl Zeiss Co., Ltd.).

### Device fabrication and characterization

The PSCs were fabricated on the PAP or ITO electrodes with the structure consisting of substrate/cathode (PAP or ITO)/PEI:BHJ/MoO_x_/Ag. The pre-cleaned ITO-coated substrates were treated with ultraviolet–ozone before device fabrication. The ITO and PAP electrodes were transferred to a glove box to spin-cast the PEI:BHJ solution. The PEI:BHJ solution was spun-cast onto the electrodes at 1,200 r.p.m. Finally, the 5-nm-thick MoO_x_ layer and 110-nm-thick Ag anode (area of 4.64 mm^2^) were deposited by thermal evaporation under a high vacuum (<10^−7^ Torr). The PLEDs were fabricated with a structure consisting of the substrate/anode (PAP or ITO/PEDOT:PSS)/P-PPV/Ca/Al. The pre-cleaned ITO-coated substrates were treated with ultraviolet–ozone before device fabrication. The PEDOT:PSS layer was coated on the ITO-coated substrates at 5,000 r.p.m. and then air dried at 100 °C for 10 min. The ITO and PAP electrodes were transferred to a glove box to spin-cast the P-PPV solution. The P-PPV solution was spun-cast onto the PAP and ITO electrodes at 2,500 r.p.m. and then dried at 80 °C for 10 min in a glove box. Finally, the 20-nm-thick Ca/80-nm-thick Al electrodes (area of 4.64 mm^2^) were deposited by thermal evaporation under a high vacuum (<10^−7^ Torr). The *J-V* characteristics of the PSCs were measured using a Keithley 236 Source Measure Unit under illumination by an Air Mass 1.5G global (AM 1.5G) with an irradiation intensity of 100 mW cm^−2^; the intensity of the spectrum of the Xenon lamp (150 W Oriel) of a solar simulator was calibrated using a calibrated standard silicon solar cell guaranteed by the National Renewable Energy Laboratory. The External quantum efficiency (EQE) data of the PSCs were obtained by a solar cell spectral response/QE/IPCE measurement system (PV Measurements, Inc.). The *J-V-L* characteristics of the PLEDs were measured using a PR650 spectrophotometer with a Keithley 2,400 Source Measure Unit. Many devices using our electrodes (over 50 devices for each electrode) were fabricated and characterized to optimize the device performance and confirm that the devices based on the electrodes are highly reproducible with high performance.

## Author contributions

H.K., Suhyun Jung, and K.L. conceived the idea. H.K. and Suhyun Jung performed the majority of the experiments. Soyeong Jeong helped with the fabrication and characterization of the electrodes and devices. G.K. performed the optical simulations. H.K., Suhyun Jung, and K.L. prepared the manuscript. K.L. guided and directed the research. All authors discussed the results and contributed to the paper.

## Additional information

**How to cite this article:** Kang, H. *et al.* Polymer-metal hybrid transparent electrodes for flexible electronics. *Nat. Commun.* 6:6503 doi: 10.1038/ncomms7503 (2015).

## Supplementary Material

Supplementary Information Supplementary Figures 1-8

## Figures and Tables

**Table 1:** Performance parameters of the PSCs and PLEDs with different substrates and electrodes.

**Substrate**	**Electrode**	**PSC parameters**					**PLED parameters**	
		***V***_**oc**_ **(V)**	***J***_**sc**_ **(mA** **cm**^−**2**^**)**	**FF**	***η*** **(%)**	***η***_**ave**_ **(%)**	***L***_**max**_ **(cd** **m**^−**2**^**)**	***C***_**max**_ **(cd** **A**^−**1**^**)**
Glass	ITO	0.78	17.03	0.73	9.7	9.5	20550	5.97
	PAP	0.79	17.28	0.74	10.1	9.9	21370	5.99
PEN	ITO	0.78	16.11	0.73	9.2	9.0	8037	5.59
	PAP	0.79	16.94	0.74	9.9	9.8	8101	6.10

FF, fill factor; ITO, indium tin oxide; PAP, PEI/Ag/PEDOT:PSS; PEN, poly(ethylene naphthalate); PLED, polymer light-emitting diode; PSC, polymer solar cell.

Average efficiency (*η*_ave_) is calculated from the efficiencies of about 10 devices fabricated on each electrode.
