# Supplementary material for: Polymer-metal hybrid transparent electrodes for flexible electronics
Source: Nat Commun. 2015 Mar 19;6:6503. doi: 10.1038/ncomms7503 (PMC4382999; doi:10.1038/ncomms7503)
Supplement: Supplementary Information — Supplementary Figures 1-8 [file ncomms7503-s1.pdf]

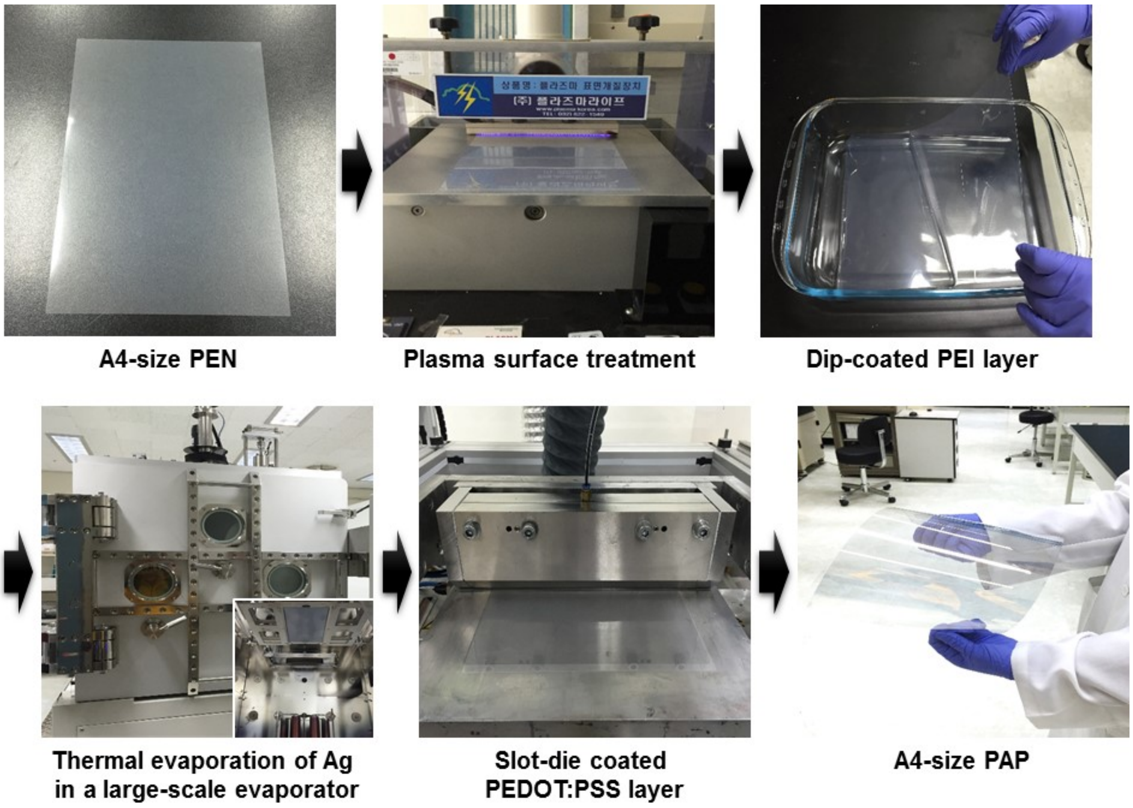

Supplementary Figure 1 | Photographs for the fabrication process of the large-area PAP. Large-area PAP electrode (A4 size) fabricated using a combination of vacuum evaporation for the Ag layer and solution-based processing for the PEI and PEDOT:PSS layers.

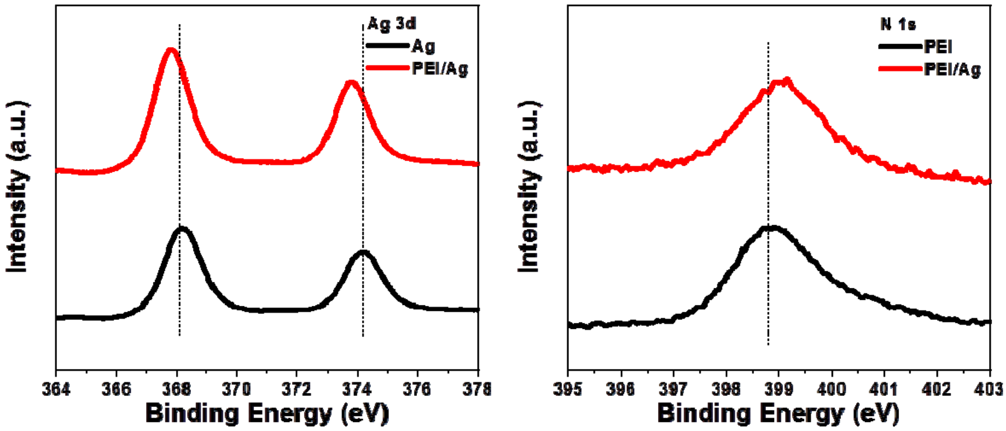

Supplementary Figure 2 | Surface analysis of the samples. High-resolution XPS spectra of Ag 3d core level (left) and N 1s core level (right) of the bare-Ag, bare PEI, and PEI/Ag samples. The high-resolution X-ray photoelectron spectroscopy (XPS) spectra of the PEI/Ag sample exhibits a negative chemical shift at the Ag 3d core level and a positive chemical shift at the N 1s core level compared with those of the bare-Ag and PEI samples. These chemical shifts indicate that the functional amines of the PEI layer offer the unshared electron pairs to the Ag atoms to form coordinate covalent bonds.

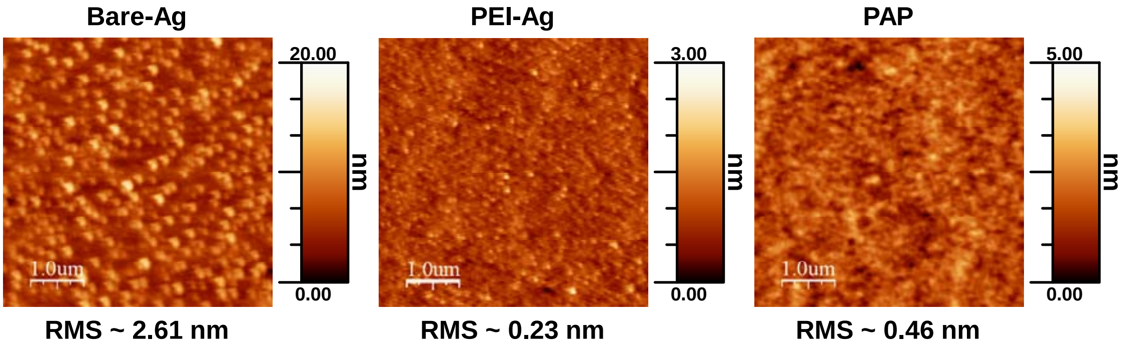

Supplementary Figure 3 | AFM topology images. AFM images of the bare-Ag, PEI-Ag and PAP on glass substrates.

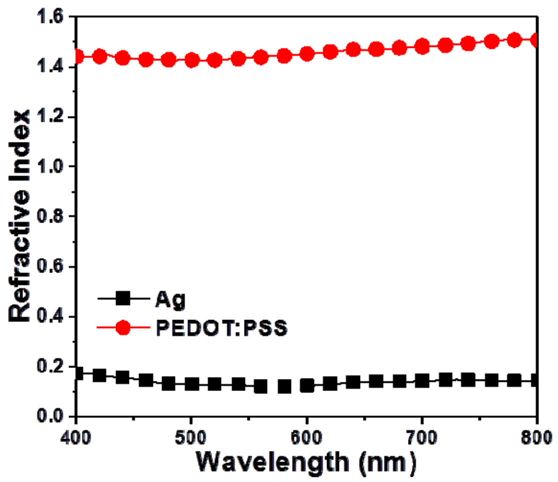

Supplementary Figure 4 | Refractive indexes of Ag and PEDOT:PSS. The large difference between the refractive indexes of Ag and PEDOT:PSS results in destructive interference that improves the transmittance of the PAP electrode.

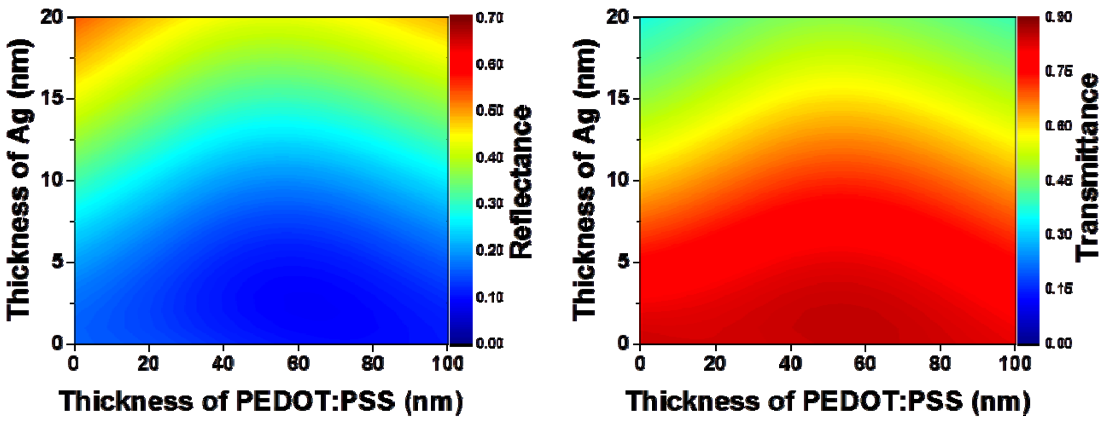

Supplementary Figure 5 | Optical simulation of the PAP electrode using a transfer matrix method. The optimum thickness of the PEDOT:PSS layer is approximately 50 nm on a 9 nm-thick Ag film

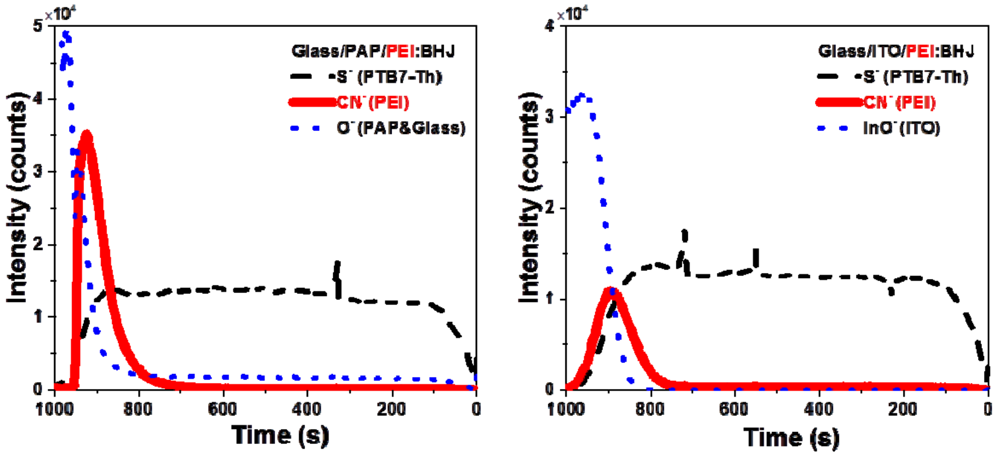

Supplementary Figure 6 | TOF-SIMS depth profiling. TOF-SIMS depth profiles of the PAP/PEI:BHJ and ITO/PEI:BHJ samples. To confirm the vertical phase separation of the PEI:BHJ blend on the electrodes, we performed time-of-flight secondary-ion mass spectrometry (TOF-SIMS). The S^-, CN^-, O^- and InO^- peaks can be assigned to PTB7-Th, PEI, PAP (PEDOT:PSS) and ITO, respectively. In both samples, the peak intensities of the S^- ions decreased near the PAP or ITO surfaces, whereas the peak intensities of the CN^- ions increased in the corresponding region. This result implies that the PEI layers were vertically self-assembled on the surfaces of the electrodes through the single-coating process of the PEI:BHJ blend.

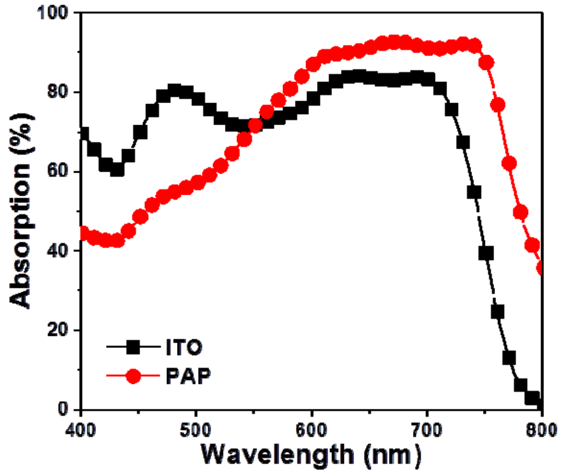

Supplementary Figure 7 | Absorption spectra (under reflection mode) of the PSCs. The absorption intensity of the PAP-based PSC is greater than that of the ITO-based PSC at longer wavelengths (550-800 nm) due to the microcavity-induced light trapping effect between the bottom PAP cathode and Ag anode.

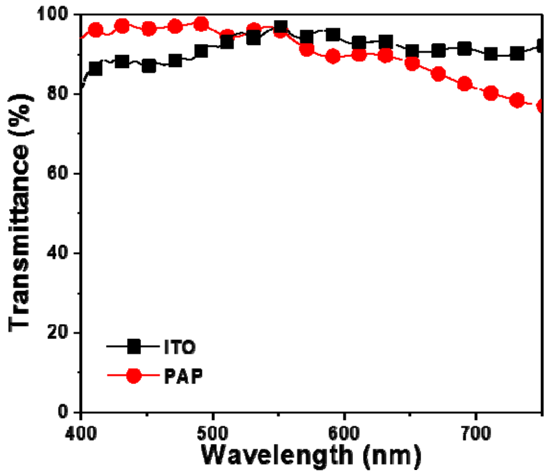

Supplementary Figure 8 | Transmittance spectra of the ITO and PAP electrodes. The PAP electrode exhibits a transmittance comparable to that of the ITO electrode in the visible range.
